# Factors affecting nurses’ childbearing intentions of nurses working in teaching hospitals in Yazd, Iran: a study based on theory of planned behavior

**DOI:** 10.1186/s12912-025-03442-w

**Published:** 2025-07-08

**Authors:** Adel Eftekhari, Tahere Sharifi, Nahid Khoddami, Mostafa Sadoghian, Somayeh Abedini, Najmeh Baghian

**Affiliations:** 1 Department of Nursing, Meybod School of Medical Sciences, Shahid Sadoughi University of Medical Sciences, Yazd, Iran; 2 Nursing Care Research Center, Semnan University of Medical Sciences, Semnan, Iran; 3 Departments of Biostatistics and Epidemiology, School of Public Health, Shahid Sadoughi University of Medical Sciences, Yazd, Iran; 4 Department of Health Management and Economics, School of Public Health, Shahid Sadoughi University of Medical Sciences, Yazd, Iran; 5 Clinical Research Development Center, Shahid Rahnemoon Hospital, Shahid Sadoughi University of Medical Sciences, Yazd, Iran; 6 Student Research Committee, Shahid Sadoughi University of Medical Sciences, Yazd, Iran

**Keywords:** Nurse, Childbearing intention, Childbearing, Theory of planned behavior

## Abstract

**Background:**

Nowadays, declining fertility rates are a significant societal challenge that particularly affects working women. While research has examined a variety of contributing factors, there is a significant lack of studies specifically on nurses, whose demanding clinical schedules and work environments may significantly influence their childbearing intentions. This study, using the theory of planned behavior, examines these factors for nurses in Yazd teaching hospitals. The results provide valuable data for policymakers to design targeted strategies and support systems to encourage nursing professionals to pursue parenting responsibilities and ultimately reduce the negative effects of low fertility rates.

**Methods:**

This cross-sectional study was conducted on female nurses working in teaching hospitals in Yazd, Iran (2024). To do so, 190 nurses were selected using stratified proportional random sampling and a 14-item questionnaire developed by Nakhaei et al. study was completed. Data were analyzed using descriptive statistics (mean and standard deviation) and inferential statistics including independent samples T-test, one-way ANOVA, Mann-Whitney test, Kruskal-Wallis, Pearson correlation coefficient, Spearman correlation coefficient, and logistic regression.

**Results:**

Based on our findings, 35% of nurses intended to have children in the next three years. Based on the results of the Pearson correlation coefficient test, a significant inverse correlation was observed between the subjective norm towards having children and the number of children (*P* < 0.001, *r*=-0.450). A statistically significant difference was reported in the mean score of perceived control over childbearing in terms of childbearing intention (*P* = 0.019); besides, a statistically significant difference was observed regarding childbearing in terms of age group (*P* = 0.004). Multivariate regression analysis with the effect of background variables considered constant, only the number of children had a significant effect on subjective norm (*P* < 0.001), meaning that for each added child, the score of subjective norm towards childbearing decreased meanly by 1.657 points.

**Conclusions:**

Given the negative impact of increasing age on perceived control and childbearing intention, health policymakers and hospital administrators can help ameliorate perceived control and foster the childbearing intention by creating more flexible working conditions and incentive policies, including financial support for female nurses. Moreover, programs that focus on encouraging childbearing in younger women can be more effective.

**Clinical trial number:**

Not applicable.

## Background

Today, population is one of the most critical economic, social and infrastructural components of any planning and policy-making process round the globe so that demographic changes can play a significant role in the economic and social development of any community. Fertility rate is one of the most important factors affecting population changes [1]. According to some reports in recent years, significant demographic changes have occurred in the world, so that we are witnessing a significant reduction in the fertility rate round the world [2]. The declined fertility began in European countries and is now being observed in Asian countries, too. According to the reported results, the global fertility rate has decreased from 3.2 live births per woman in 1990 to 2.5 live births in 2019 [3]. Iran is no exception in this regard, as according to the World Bank, Iran’s population growth rate will fall below 1% by 2025 [4]. The declined fertility rate and the consequences it will bring about in various fields, including aging and socioeconomic issues, have prompted many countries around the world to reconsider their population control policies and strengthen the childbearing incentive in their communities through different methods. Childbearing policies have been one of the Iranian government’s top priorities in recent years. Although childbearing and birth seem simple, it is a complex issue that is influenced by numerous quantitative and qualitative factors, including women’s education level, occupation, family income, gender norms and values, culture, ethnicity, and religion [5]. Some important issues raised in the field of humanities include paying attention to the relationship between women’s employment in the labor market as one of the important and influential factors in economic growth, and fertility rate as an influential factor in population growth, as well as the combined effect of both variables on the position and status of women. Additionally, the duration of women’s work is known to be an important factor in the childbearing behavior of working women [6]. Planning and decision-making about childbearing is not performed only by women and requires examining the economic, social, cultural, and demographic characteristics of parents. Factors such as the expansion of urbanization, women’s increased education level, and the subsequent increase in women’s employment, as well as the increased age of marriage, have reduced the childbearing intention among Iranian women, so that the fertility rate has been on a downward course in the past two decades [6]. The stressful conditions prevailing in medical centers and the employment of nurses in inpatient wards and their presence in frequent and rotating shifts have been proven in various studies, and their employment in the clinic can also be considered as one of the possible influential causes in childbearing decisions. Women working in healthcare centers, similar to other organizations, face a dual role in employment. On the one hand, they are responsible for household chores, including raising children and marital issues; on the other hand, they are responsible for clinical duties as an employee and working woman [7, 8]. One of the most effective theories of health education in fertility intention is the theory of planned behavior (TPB). It is also one of the important theories in explaining behaviors and their etiology in behavioral and social sciences. According to this theory, the most important determinant of a person’s behavior is behavioral intention; then, a combination of attitude towards performing the behavior, abstract norms, and perceived behavioral control can lead to performing a behavior. The use of TPB to examine nurses’ childbearing intentions is because this theory can provide a better prediction of actual childbearing intentions and behaviors by taking into account the individual’s attitude towards childbearing, perceived social norms surrounding it, and perceived behavioral control. TPB examines the various factors that influence nurses’ decisions about childbearing and allows targeted intervention programs to be designed to promote or improve these intentions and behaviors [9]. The aging population, declining birth rates, and the increasing need for nursing professionals underscore the importance of identifying factors affecting nurses’ childbearing intentions. Understanding these factors can not only help predict and manage the shortage of human resources, but can also lead to the creation of supportive strategies and improve nurses’ quality of life and job satisfaction. The theory of planned behavior, considering the mental and social factors affecting decision-making, can help to understand this issue more deeply and provide solutions to increase the intention to have children in this occupational group. By examining the relationship between attitude, perceived norm, and perceived behavioral control with nurses’ childbearing intentions, this study can lead to the provision of targeted and effective interventions to improve the nursing human resources and ultimately improve the quality of healthcare services.

## Methods

This analytical cross-sectional study was carried out in 2024 on nurses working in teaching hospitals in Yazd (Shahid Sadoughi, Shahid Rahnemoon and Afshar). A total of 190 nurses were selected as the sample using stratified random sampling based on the results of the pilot study (30 nurses completed the questionnaire) and considering a standard deviation of 53%, an error of 8%, a confidence interval of 95%, and attrition rate of 10%. The inclusion criterion of the study was married nurses aged 15 to 45 years. Thus, first the teaching hospitals of Yazd city were considered as different classes and then, according to the number of nurses in each hospital, the samples were selected proportionally and randomly from each center. The exclusion criterion was Being infertile and unwillingness to participate in the study. The instrument used was a questionnaire developed by Nakhaei et al. in their study entitled: “Development and Validation of the Questionnaire of Factors Affecting Childbearing Intention in Iranian Women based on the Theory of Planned Behavior”. The three components of this questionnaire include attitude (8 items), subjective norm (3 items), and perceived control (3 items). The factor loadings of all three constructs were above the minimum acceptable value of 0.4. The Cronbach’s α of the three constructs were 0.83, 0.79, and 0.66, respectively. The measurement scale is based on a five-point Likert scale, ranging from very low (1 point) to very high (5 points) (22). SPSS02_27_ software was used to analyze the data. The normality of quantitative variables was examined using the Kolmogorov-Smirnov test. Independent sample T-test, one-way ANOVA, and Pearson correlation coefficient were used for normally distributed variables. If the variables were not normally distributed, corresponding nonparametric tests of Mann-Whitney, Kruskal-Wallis, and Spearman correlation coefficient were used. Chi-square test was used to examine the relationship between nurses’ childbearing intention and demographic information (Age group, Education level, Work experience, Work unit, Work shift, Employment status, Accommodation status, Income rate, Number of children). Multivariate logistic regression was also used to examine the factors affecting nurses’ childbearing intention (*P* = 0.05).

## Results

Based on the findings, 190 female nurses working in Yazd teaching hospitals (Shahid Sadoughi, Shahid Rahnemoon, and Afshar) who were eligible based on the inclusion criteria participated in the study. Totally, 183 completed questionnaires were returned by nurses, indicating that the response rate to the questionnaires was 96.3%. The educational level of 11 nurses (6.0%) was diploma or associate diploma, 151 nurses (82.5%) had bachelor’s degree, and 21 nurses (11.5%) had master’s degree or higher. Besides, 99 nurses (54.1%) had more than 10 years of work experience and the rest had less than 10 years. Moreover, 78 nurses (42.6%) were working in clinical ward and the rest in other units. Most nurses worked on rotating shifts (71.6%) and on a contract-formal employment basis (80.9%). The majority owned their houses, had an income of 100–200 million Rials (79.2%), and had 2 children (45.9%). The mean score of childbearing intention was calculated to be 35%. Based on the results of the Spearman correlation coefficient, a significant inverse correlation was observed between the score of the subjective norm towards childbearing and the number of children (*P* < 0.001, *r*=-0.450). In other words, women with more children had lower subjective norms (feeling pressure from others who emphasized having children). A statistically significant difference was observed in the mean score of the perceived control towards childbearing in terms of childbearing intention (*P* = 0.019). This means that the mean perceived control score of nurses who intended to have children was significantly higher than that of nurses who did not intend to have children. Childbearing intention exerted a weak effect on the score of perceived control towards childbearing (Table 1).

**Table 1:** Mean score of childbearing intention and factors affecting childbearing intention in nurses

Component		Frequency	Mean ± SD	Effect size	*P*-value
Attitude	Yes	64	23.92 ± 6.94	*0.061	#0.693
	No	119	23.49 ± 7.16		
Subjective norm	Yes	64	8.12 ± 3.58	*0.087	##0.552
	No	119	7.77 ± 3.64		
Perceived control	Yes	64	12.51 ± 2.76	*0.347	##0.019
	No	119	11.27 ± 3.48		

* Cohen’s d

# Independent sample t-test

##Mann-Whitney U Test

Based on the results displayed in Table 2, a statistically significant difference was observed in the mean score of the attitude towards childbearing by employment unit (*P* = 0.039). Based on the results of the *post hoc* test, the mean attitude score of nurses working in the administrative-financial unit was significantly higher than that of nurses working in the ICU(*P* = 0.029). Employment unit had an approximately medium-size effect on the score of the attitude towards childbearing. Further, the mean attitude score of house tenant nurses was significantly lower than that of house owners (*P* = 0.051) and others (*P* = 0.005). Housing status had an approximately medium-size effect on the score of the attitude towards childbearing. A significant inverse correlation was observed between the score of the subjective norm towards childbearing and the number of children (*P* < 0.001, *r*=-0.450). In other words, people with more children had lower subjective norm (feeling pressure from others who emphasized childbearing). A statistically significant difference was observed in the mean score of the subjective norm towards childbearing in terms of age group (*P* = 0.004), meaning that the mean subjective norm score of nurses aged 23–34 years was significantly higher than that of nurses aged 35–45 years. Age had an approximately medium-size effect on the score of the subjective norm towards childbearing (Table 3). A statistically significant difference was observed in the mean score of the subjective norm towards childbearing in terms of work experience (*P* < 0.001). Work experience exerted a moderate effect on the score of the subjective norm towards childbearing. Furthermore, a statistically significant difference was observed in the mean score of the perceived control towards childbearing in terms of childbearing intention (*P* = 0.019); also, a statistically significant difference was observed towards childbearing in terms of age group (*P* = 0.004). Also, a statistically significant difference was observed in the mean score of the perceived control towards childbearing according to shift type (*P* = 0.011), suggesting that the mean score of perceived control of rotating shift nurses was significantly higher than that of fixed shift nurses.

**Table 2:** Determining and comparing the mean scores of attitude, subjective norm, and perceived control towards childbearing in nurses in terms of childbearing intention and demographic variables

Demographic variables Components		f	Mean ± SD	Effect size	*P*-value	Mean ± SD	Effect size	*P*-value	Mean ± SD	Effect size	*P*-value
Childbearing intention	Yes	64	23.92 ± 6.94	0.061	0.693	8.12 ± 3.58	0.087	0.552	12.51 ± 2.76	0.347	0.019
	**±**No	119	23.49 ± 7.16			7.77 ± 3.64			11.27 ± 3.48		
Age group	23–34 years	78	23.79 ± 6.91	0.038	0.798	8.79 ± 3.52	0.436	0.004	12.67 ± 2.30	0.419	0.004
	35–45 years	105	23.52 ± 7.21			7.22 ± 3.56			11.00 ± 3.72		
Education level	Diploma & associate diploma	11	24.54 ± 9.50	0.001	0.906	8.36 ± 3.29	0.003	0.568	10.45 ± 3.14	0.018	0.070
	BS	151	23.56 ± 7.01			7.77 ± 3.67			11.90 ± 3.33		
	MSc & higher	21	23.71 ± 6.32			8.57 ± 3.47			10.95 ± 2.95		
Work experience	< 5 years	32	24.00 ± 7.80	0.009	0.463	10.28 ± 2.80	0.102	< 0.001	12.31 ± 2.19	0.016	0.091
	5–10 years	52	24.51 ± 7.36			8.15 ± 3.49			12.42 ± 2.91		
	> 10 years	99	23.06 ± 6.67			6.70 ± 3.57			11.14 ± 3.67		
Work unit	Inpatient wards	78	23.24 ± 6.53	0.055	0.039	7.92 ± 3.50	0.009	0.662	11.39 ± 3.31	0.011	0.201
	ICUs	41	21.85 ± 7.90			7.21 ± 3.73			11.41 ± 3.74		
	Official-financial	29	26.82 ± 6.21			8.37 ± 3.60			11.75 ± 2.81		
	ER	19	22.78 ± 7.14			8.42 ± 3.81			13.10 ± 2.94		
	OR	16	25.37 ± 7.38			8.00 ± 3.88			12.25 ± 3.06		
Work shift	Fixed	52	24.71 ± 5.94	0.212	0.156	7.86 ± 3.58	0.011	0.938	10.83 ± 3.35	0.375	0.011
	Rotational	131	23.21 ± 7.45			7.90 ± 3.64			12.06 ± 3.21		
Employment status	Firm contract	13	23.92 ± 9.55	0.008	0.678	8.92 ± 2.69	0.019	0.093	11.69 ± 2.92	0.007	0.853
	Due commitment	13	22.84 ± 7.36			9.84 ± 2.44			12.84 ± 1.62		
	Contractual-formal	148	23.52 ± 6.90			7.68 ± 3.73			11.61 ± 3.42		
	Others	9	26.33 ± 5.85			7.11 ± 3.48			11.66 ± 3.42		
Accommodation status	Tenant	36	20.91 ± 7.51	0.058	0.005	8.58 ± 3.69	0.014	0.101	12.27 ± 3.38	0.004	0.258
	Owner	135	23.96 ± 6.71			7.57 ± 3.57			11.52 ± 3.28		
	Others	12	28.16 ± 7.12			9.50 ± 3.45			12.08 ± 3.14		
Income rate	< 100,000,000 Rials	27	23.11 ± 7.86	0.007	0.549	8.66 ± 3.67	0.003	0.489	11.22 ± 3.74	0.002	0.305
	100,000,000–200,000,000 Rials	145	23.57 ± 6.94			7.75 ± 3.50			11.90 ± 3.15		
	> 200,000,000 Rials	11	25.81 ± 6.95			7.81 ± 4.97			10.36 ± 3.82		
Number of children	----	----	**----**	0.105	0.157	----	-0.450	< 0.001	----	-0.007	0.157

**Table 3:** Investigating factors affecting the childbearing intention in nurses

Variable	Unstandardized coefficients		Odds ratio	IC=%95		*P*-value
	(β)	Standard error		Minimum	Maximum	
Age (35–45 years)	2.397	0.626	10.985	3.222	37.449	< 0.001
Work experience (> 10 years)	**Reference**					
Work experience (5–10 years)	-0.008	0.718	0.992	0.243	4.050	0.991
Work experience (< 5 years)	-0.300	0.643	0.741	0.214	2.566	0.636
Work shift type (rotational)	-0.331	0.467	0.718	0.288	1.793	0.478
Employment status (contractual-formal)	-0.304	0.488	0.738	0.283	1.922	0.534
Accommodation status (others)	**Reference**					
Accommodation status (owners)	-0.063	0.776	0.939	0.205	4.298	0.935
Accommodation status (tenants)	-0.320	0.712	0.726	0.180	2.932	0.653
Perceived control	0.049	0.065	1.051	0.925	1.193	0.447

Based on the results of the bivariate tests given in Table 3, variables that had a significant relationship with the childbearing intention were entered into the regression model to examine their simultaneous effect on the childbearing intention. The only variable significantly associated with the childbearing intention was age (*P* < 0.001). In other words, the chance of intending to have children in nurses less than 35 years of age was 10.985 times higher than that in nurses over 35 years of age.

## Discussion

The present study assessed the factors influencing the childbearing intention among nurses working in teaching hospitals in Yazd based on the theory of planned behavior. This theory can appropriately examine the decision-making process regarding childbearing among nurses via emphasizing the three main components of attitude, subjective norms, and perceived behavioral control. The findings suggested that 35% of nurses intended to have children within the next three years. Given that the target population of the present study is employed women with nursing education, studies conducted in other Middle Eastern countries, including Pakistan [10] and Egypt [11], report similar results indicating a decrease in women’s intention to have children with increasing levels of education and employment. In different results, research in Saudi Arabia [12] showed that 80% of female medical students intend to have children in the near future. Such a difference in results could be due to the difference in the average age of the target population in the two studies (students with a lower average age than employed nurses). The low childbearing intentions of this group of women further underscore the need for corrective measures. One of these interventions could be education and cultural development in community, as intervention studies have shown that education can exert a positive impact on women’s fertility intentions [1]. Hence, it is recommended to increase public awareness about the individual and social benefits of having children through mass media and to strengthen relevant educational programs in schools and universities. Nonetheless, its impact is not limited to educational factors; rather, social, cultural, and economic factors must also be considered. Contrary to some previous evidence in Iran, Norway, Italy, and Romania suggesting that women’s subjective norm [1–5] and attitude [1, 4, 5] can play a key role in fertility intention, the present findings indicated that there was no significant relationship between the childbearing intention and the two components of attitude and subjective norm. Similarly, in another review study, attitudes toward childbearing had the greatest impact on the childbearing intention in three countries: Russia, Italy, and Hungary; the dominant issues for couples seemed to be mainly related to their beliefs about whether having children would make their lives better or worse [2]. The reason for such a difference in results can be attributed to the characteristics of the target group. This is because in the present study, unlike the aforementioned studies, female nurses working in hospitals were studied, the majority of whom had academic education and were all over 23 years old. These issues have resulted in the ineffectiveness of other people’s expectations and attitudes towards childbearing through increased experience and awareness, understanding more complex issues such as paying attention to the effects of physical and mental health, economic status, work status, desire to continue education, etc. Consequently, the findings revealed that childbearing intentions are mainly determined by the individual’s perceived control, rather than by subjective norms (others’ expectations) and attitudes. Nevertheless, in the regression model, this effect disappeared when we controlled for contextual and demographic variables. This suggests that the effect of perceived control on having children differs significantly from personal circumstances. In contrast, similarly, in two countries, Russia and France, perceived control exerted a statistically significant effect on intention [2]. In a study by Williamson et al. in Canada, attitudes, subjective norms, and perceived control all significantly accounted for 61% of the variance in the intention to delay childbearing at age 30 [6]. Among the reasons for the inconsistency between the results are differences in the income status of the target group, the instrument used, and demographic characteristics such as the age groups of the participants. Among the contextual variables, the mean score of nurses’ attitude working in the administrative-financial unit was significantly higher than that of nurses working in the ICU (*P* = 0.029). Existing evidence suggests that ICU nurses are more likely to experience work-family conflict due to specific work conditions such as the responsibility of caring for critically-ill patients, emotional burden, and frequent exposure to emotional situations such as giving bad news to patients’ families and the resulting psychological stress [7, 8]. Thus, they are likely to have a more negative attitude towards childbearing compared to nurses working in non-clinical units who do not experience such stressful situations, as pregnancy and a new child will complicate the situation and exacerbate work-family conflict. So, one of the positive measures in this regard would be to develop and implement policies to support nurses with children, such as flexible work shifts, etc. Consistent with the results, in a multivariate regression analysis, the attitude of those who chose their housing status as other or owner was 3.21 points higher than those who were tenants. According to the findings by Matera et al., perceived economic uncertainty affects couples’ attitudes towards childbearing [9]. The relationship between housing and fertility has been documented in the global literature, and housing ownership or tenure has been identified as one of the main mechanisms influencing childbearing [1]. Studies in Iran [13] and Sweden [14] have pointed to housing as one of the reasons for couples’ economic anxiety and fear, and consider it to be effective in couples’ childbearing. As a result, one of the government’s measures to induce a positive attitude in couples, especially working people, and increase the childbearing intention can be to provide long-term housing facilities and provide affordable housing. Based on the findings, in a multivariate regression analysis with the effect of background variables considered as constant, only the number of children exerted a significant effect on subjective norm (*P* < 0.001), indicating that for each additional child, the score of subjective norm towards childbearing decreased by an mean of 1.657 points. Ajzen et al. also found in their study that one of the important background factors in fertility research is the number of children that affects the opinions of others about childbearing (subjective norms) [2]. In particular, the decision to have a first child is qualitatively different from the decision to have a second or subsequent child and, in fact, the decision to have a second child is more complex. Given that in psychologists’ and economists’ viewpoint, having a more productive economic society [15] and having healthier children in terms of mental health and resilience, hopefulness, and optimism [16] are the effects of having more than one child, thus, one of the most important measures to be taken is to create a culture of “more children, more dynamic community” by raising awareness among the community about the necessity of transitioning from childless and single-child families to larger families. Based on the results, in a multivariate regression analysis, for every year of age, the score of perceived control towards childbearing decreased by a mean of 0.057 points. A review study found conflicting results, showing that in French women, older age is associated with stronger perceptions of control [2]. It appears that the difference in cultural, social, and economic conditions of the target groups in the two studies is the cause of such a disparity in results.

For example, in France [17], government policies provide extensive financial and structural support for working mothers, including publicly subsidized childcare services, substantial maternity and parental leave, and workplace protection laws that prevent occupational harm related to childbearing. This can enhance women’s sense of control and autonomy in reproductive decisions. In contrast, in Iran [18], despite policies to encourage childbearing, many women still face a lack of institutional support; these constraints, combined with prevailing cultural expectations about women’s roles in childrearing, can reduce women’s perceived control as they balance their work and family responsibilities.

Low perceived control indicates a high impact of physical and mental health, and economic, educational, and work status on the decision to have children. Younger women have more control over their childbearing decisions due to better physical health, more stable economic and career conditions, and greater hope of managing work and family responsibilities simultaneously. However, physical and psychological problems, financial constraints, and job obligations increase with age, and this can be an obstacle to having children. On the other hand, based on available evidence, confidence in the length of reproductive life is the main factor in perceived control [6]. For this reason, with increasing age and decreasing confidence in the duration of reproductive life, perceived control also decreases. Based on the results of the multiple regression model, only age can exert a significant effect on determining individual reproductive intention (*P* < 001); in other words, the chance of intending to have children in nurses under 35 years of age is 10.985 times higher than in nurses over 35 years of age. This finding is also consistent with the existing evidence regarding a significant relationship between age and reproductive intention [4, 19, 20]. For example, in the study by Araban et al., multivariate regression analysis showed that a number of variables, including age, were important factors associated with pregnancy intention [4]. Furthermore, another study (2020) among Chinese women following China’s new global two-child policy demonstrated by regression that the age range of 25–39 years, along with some economic and social factors, was associated with a greater intention to have a second child (*P* < 0.05) [21]. In contradictory results, a study by Modiri et al. (2021) using multivariate tests showed that socio-economic characteristics of Tehrani men, such as education, income, parents’ socioeconomic status, and employment status, were negatively associated with their childbearing intention [22]. Contrary to the results of the present study, age was not introduced as an influential factor in this study. The reason for these contradictory results can be attributed to the gender of the target group in the two studies, because, unlike women, age of men is not an influential factor in their fertility and, consequently, their intention to reproduce. Yet, as women age, issues related to high-risk pregnancies and decreased natural fertility become serious concerns, thence exerting a negative impact on the childbearing intention. A statistically significant relationship was observed between the childbearing intention and the type of shift and employment status; the majority of nurses (80.9%) with a formal-contractual employment status had the childbearing intention. Similarly, findings by Araban et al. [4] in Iran and a review study [23] showed that individuals’ employment status and job characteristics (including shift work) were significantly associated with their childbearing intentions, respectively. One of the notable points in the results of this study was the statistically significant relationship between the childbearing intention, housing, and employment status (*P* = 0.040), so that most nurses who owned a home intended to have children (62.5%). Similarly, other research in Saudi Arabia, Egypt, Iran, and a review of similar studies conducted around the world showed that economic factors such as housing status, job status, and employment are important factors influencing childbearing [1, 4, 13, 20, 23]. The findings of other studies showed that the country’s economic uncertainty, including the housing situation, may discourage women from planning for pregnancy [2, 9]. This is because it seems that the stable status of formal and contractual employment and owning a house will increase couples’ childbearing intention by creating security and peace of mind regarding the provision of living expenses and shelter. Finally, by improving employment policies in the country to create job security and provide long-term housing facilities, the path can be paved for increasing the childbearing intention.

### Strengths and weaknesses

As part of the health community, nurses have specific occupational and professional circumstances that may influence family decisions such as childbearing. Given that nurses account for more than 60% of healthcare workers, the specific focus on this group makes the study unique and can have practical and meaningful results for health policymakers. In particular, this study indirectly examined the relationship between professional life and personal decisions such as childbearing. This is of psychosocial importance because it shows how workplace conditions influence individuals’ personal intentions and decisions regarding childbearing.

### Limitations of the study

Although the present study adds to the body of knowledge regarding nurses’ decision-making regarding childbearing, the differences in characteristics of nurses working in hospitals compared to other occupational groups and other geographical regions should be considered when generalizing the results. Besides, the present study was conducted cross-sectionally; so, conducting longitudinal and interventional research in this area will help provide a broader perspective on the childbearing intention among working women and the impact of education and culture on their improvement.

## Conclusions

According to the findings of this study, age plays a significant role in decisions related to childbearing. The childbearing intention in women under 35 years of age is about 11 times higher than that of older women. The findings of this study can be useful for health decision-makers, hospital managers, and policymakers in designing supportive policies for nurses and encouraging them to have children. Given the negative impact of increasing age on perceived control and childbearing intention, health policymakers and hospital managers can help improve perceived control and increase the childbearing intention by creating facilities such as more flexible working conditions and financial support for female nurses. Further, programs that focus on encouraging childbearing among younger women may be more effective. These include housing incentives, support for working parents, and better work-life balance. As a result, governments can increase the likelihood of childbearing behavior by providing and facilitating the conditions for marriage and childbearing, especially for those under the age of 35, in terms of social, cultural, and economic conditions. Finally, the present study also examined various psychological and social factors affecting decision-making regarding childbearing. This multifaceted analysis helps to gain a more complete understanding of nurses’ decision-making process.

## Data Availability

All generated and analyzed data in this research are included in this manuscript. The datasets used and/or analyzed during the current study are available from the corresponding author upon reasonable request.
